# Features of combined gut bacteria and fungi from a Chinese cohort of colorectal cancer, colorectal adenoma, and post-operative patients

**DOI:** 10.3389/fmicb.2023.1236583

**Published:** 2023-08-08

**Authors:** Xiaopeng Li, Jiahui Feng, Zhanggui Wang, Gang Liu, Fan Wang

**Affiliations:** ^1^Department of Radiation Oncology, The First Affiliated Hospital of Anhui Medical University, Hefei, China; ^2^Department of Radiation Oncology, Anhui No. 2 Provincial People's Hospital, Hefei, China; ^3^School of Life Sciences, Anhui Medical University, Hefei, China

**Keywords:** colorectal cancer, colorectal adenomas, feces, biomarker, OTUs

## Abstract

Colorectal cancer (CRC) accounts for the third highest morbidity burden among malignant tumors worldwide. Previous studies investigated gut microbiome changes that occur during colorectal adenomas (CRA) progression to overt CRC, thus highlighting the importance of the gut microbiome in carcinogenesis. However, few studies have examined gut microbiome characteristics across the entire spectrum, from CRC development to treatment. The study used 16S ribosomal ribonucleic acid and internal transcribed spacer amplicon sequencing to compare the composition of gut bacteria and fungi in a Chinese cohort of healthy controls (HC), CRC patients, CRA patients, and CRC postoperative patients (PP). Our analysis showed that beta diversity was significantly different among the four groups based on the gut bacterial and fungal data. A total of 51 species of bacteria and 8 species of fungi were identified in the HC, CRA, CRC, and PP groups. Correlation networks for both the gut bacteria and fungi in HC vs. CRA, HC vs. CRC, and HC vs. PP indicated some hub bacterial and fungal genera in each model, and the correlation between bacterial and fungal data indicated that a highly significant negative correlation exists among groups. Quantitative polymerase chain reaction (qPCR) analysis in a large cohort of HC, CRC, CRA, and PP patients demonstrated a significantly increasing trend of *Fusobacterium nucleatum, Bifidobacterium bifidum, Candida albicans*, and *Saccharomyces cerevisiae* in the feces of CRC patients than that of HC patients (*p* < 0.01). However, the abundance levels of CRA and PP were significantly lower in HC patients than those in CRC patients. Further studies are required to identify the functional consequences of the altered bacterial/fungal composition on metabolism and CRC tumorigenesis in the host.

## Introduction

Colorectal cancer (CRC) is a common malignancy of the colon and rectum, accounting for approximately 10% of all new cancer cases globally. Moreover, it carries the third highest burden of morbidity among all malignant tumors worldwide (Wong and Yu, 2019; Yang et al., 2019). In China, CRC is the second most common malignancy, with a notably rapid increase in its incidence in recent years (Thanikachalam and Khan, 2019; Guo et al., 2021). The pathogenesis of CRC includes chronic inflammation and the accumulation of genetic, epigenetic, dietary, and environmental factors; however, the exact etiology of CRC remains underexplored (Song et al., 2015; Plummer et al., 2016; Vacante et al., 2020). Colorectal adenomas (CRA) are defined as benign tumors derived from the colorectal mucosa, with the transformation process referred to as the adenoma-carcinoma sequence. Approximately 60–90% of sporadic CRCs start as premalignant lesions known as CRAs (Kim et al., 2020; Vacante et al., 2020). Surgery, radiation therapy, and chemotherapy remain the main therapeutic strategies, with an average 5-year overall survival rate of approximately 40% (Aguiar et al., 2020; Lohsiriwat et al., 2021). Despite the availability of various methods to screen for CRC, colonoscopy remains the gold standard for accurate diagnosis. However, colonoscopy's invasive and unpleasant nature often causes patients to experience unwanted pain and discomfort, leading to more than half of the preference for non-invasive screening methods.

Despite improvements in imaging technologies, the accurate diagnosis of CRC remains a clinical challenge, and over the past few decades, the gut microbiome has been preferred as a molecular biomarker and non-invasive screening method in humans (Marx, 2014; Sinha et al., 2016; Flemer et al., 2017). The current belief is that gut microbiota dysbiosis and a subsequent inappropriate or altered immune response confer a predisposition to chronic inflammation, which is known to contribute to the development of diseases, including cancer. Several studies have investigated the roles of changes in the gut microbiome in the development of adenomas and carcinomas, highlighting the impact of this process on carcinogenesis (Fearon and Carethers, 2015; Sun et al., 2016; Alhinai et al., 2019; Wong and Yu, 2019; Kim et al., 2020). Indeed, the gut microbiota has recently emerged as a central player linking various risk factors to CRC pathogenesis, and many studies have investigated the changes and its role in adenoma and carcinoma development, highlighting the impact of the gut microbiota on the development of CRA and the subsequent progression to CRC (Round and Mazmanian, 2009; Collins et al., 2011; Kamada et al., 2013; Rooks and Garrett, 2016; Gao et al., 2017; Pickard et al., 2017; Yoshii et al., 2019; Zhang et al., 2019; Kim et al., 2020). Several clinical studies have identified that the abundance and structure of gut microbiota are significantly different between patients with CRA and healthy individuals (Shen et al., 2010; Sanapareddy et al., 2012; Lu et al., 2016). Moreover, it has been reported that patients with CRC have distinct qualitative differences in their gut microbiota compared to healthy controls (HC). Patients with CRC also present with changes in microbial composition, function, and ecology (Chen et al., 2013; Louis et al., 2014; Drewes et al., 2016; Marchesi et al., 2016; Peters et al., 2016; Hibberd et al., 2017; Murphy et al., 2021). Investigations of the impact of CRA endoscopic surgery on the intestinal flora revealed that despite no alterations in the overall microbiome structure after CRA excision, the protective microbiota demonstrated an ascending trend, whereas tumor-associated microbiota exhibited a declining trend (Yu et al., 2017). Thus, the findings of these and other studies suggest that gut microbiota play complex and key roles in CRA and CRC. There is accumulating evidence that the etiology of CRC/CRA is related to the gut microbiota and that gut microbiota composition is a major risk factor for CRC and CRA (Hibberd et al., 2017; Yu et al., 2017; Murphy et al., 2021).

A considerable number of studies have suggested *Fusobacterium nucleatum* (*Fn*), *Bifidobacterium bifidum* (*Bb*), *Candida albicans*, and *Saccharomyces cerevisiae* as potential markers for CRC detection (Sokol et al., 2017; Yu et al., 2017). *Fn* and *Bb* are important gut bacteria in humans; *Fn* has been suggested by a considerable number of studies as a potential marker for CRC detection, and the abundance of this species was significantly increased in CRA and CRC (Yu et al., 2017). *Bb* typically represents the most abundant bacteria in healthy humans, supporting its specific adaptation to the human gut and its implications in terms of supporting host health. Moreover, *Bb* is one of a few probiotic strains that are effective in the treatment of gastrointestinal cancer and its symptoms, and the abundance ratio of *Fn*/*Bb* might favor the progression of CRC (Andresen et al., 2020). Fungal dysbiosis is known to play a role in the development of CRC and is characterized by decreased community diversity in addition to a higher abundance of detrimental fungi, such as *C. albicans* and *S. cerevisiae. C. albicans* and *S. cerevisiae* were also revealed to have a close association with gastrointestinal disturbances, and fungal internal transcribed spacer 2 (ITS2) library sequencing revealed the abundance of *S. cerevisiae* decreased, while that of *C. albicans* increased in inflammatory bowel disease (IBD); a shift in the gut microbiota environment was demonstrated by analyzing the correlation between bacteria and fungi (Sokol et al., 2017).

Previous studies have revealed that shifts in gut microbiota may play an important role in the initiation and progression of CRC; however, only a few studies have focused on the “biomarker” characteristics of the gut microbiota during the development of CRC and the treatment process. Therefore, a better understanding of the role of alterations in the gut microbiota is urgently needed to improve diagnostic, therapeutic, and prognostic strategies against CRC. This study aimed to explore the combined data of gut bacterial and fungal profiles in the initiation, progression, and prognosis of CRC and to further screen microbial biomarkers associated with CRC. We profiled the combined data of bacterial and fungal communities of feces in CRC, CRA, postoperative patients (PP), and HC to identify biomarker microbiomes using high-throughput sequencing of the 16S ribosomal ribonucleic acid (rRNA) and ITS gene regions. We also combined multiple data points on bacteria and fungi in CRC and used them for correlation analyses. In addition, we detected variations in the abundance of *Fn, Bb, C. albicans*, and *S. cerevisiae* in an independent large cohort of patients with CRC, CRA, PP, and HC using quantitative polymerase chain reaction (qPCR). Through these efforts, we aimed to investigate the different gut bacterial and fungal profiles among CRC, CRA, and HC to identify the marker microbiota that likely contributes to CRC development and impact treatment progression.

## Materials and methods

### Ethics statement

Written oral consent was obtained from each participant before sample collection. All of the methods were performed in accordance with relevant guidelines and regulations, including any relevant details. Informed consent was obtained from all the patients, and the study was approved by the Institutional Review Board of Anhui No. 2 Provincial People's Hospital.

### Study patients

The clinical phenotype was determined by endoscopic and pathological diagnoses. Patients with no abnormalities on colonoscopy were included in the HC group. Additionally, CRA and CRC were diagnosed according to both clinical and pathological criteria, and all CRC subjects had intact colonic lesions at the time of stool collection. Patients who had undergone CRC radical surgery with regular follow-up within 1 month to 3 years after surgery and without relapse were included in the PP group. The inclusion criteria for the four groups were as follows: (a) all participants were older than 40 years at the time of sample collection. (b) Diagnosis of CRC/CRA was defined according to clinical, radiological, endoscopic, and histological criteria and without other diseases. The tumor, node, and metastasis (TNM) classification system was used for staging patients with CRC as having TNM stage II/III disease. (c) None of the patients or HC were treated with antibiotics, colon-cleansing products, or hormones within 1 month. (d) All of the participants had no history of uninterested tract neoplasia or upper gastrointestinal tract surgery. (e) No eating habit changes in the last 4 weeks and no active gastrointestinal tract bleeding in the last 6 months.

### Sample collection

A total of 68 fecal samples were collected from 15 CRC patients, 19 CRA patients, 19 PP, and 15 HC for combined gut bacterial and fungal analyses (Supplementary Table 1). An independent cohort of 402 patients consisting of 92 HC patients, 119 CRC patients, 95 CRA patients, and 96 PP was used to detect the abundance variation of *Fn, Bb, C. albicans*, and *S. cerevisiae* (Supplementary Table 1). All fecal samples were collected at Anhui No. 2 Provincial People's Hospital and the First Affiliated Hospital of Anhui Medical University, China. All patients were asked to maintain a steady diet and lifestyle and to leave fecal samples (>0.5 g) in a germ-free containment. All fresh fecal samples were collected from the patients and placed in a sterile box, which was immediately transported to the lab on ice. All the fecal samples were collected and stored at −20°C within 4 h and −80°C within 24 h for long-term storage.

### DNA extraction, amplification, high sequencing, and qPCR analysis

Microbiota sequencing and data analysis are presented in Supplementary Figure 1. Fecal samples (50–100 mg) were weighed using a 2.0 ml centrifuge tube containing glass beads (200 mg) on ice, and deoxyribonucleic acid (DNA) was isolated from fecal samples using a MagPure Soil DNA LQ Kit (Magen, Guangdong, China) according to the manufacturer's instructions. The extracted DNA was diluted to 1 ng/μl and used as the template for PCR amplification. 343F/798R (343F: 5′-TAC GGR AGG CAG CAG-3′; 798R: 5′-AGG GTA TCT AAT CCT-3′) was used to amplify the 16S rRNA gene of gut bacteria, and ITS primers (ITS1F: 5′-CTT GGT CAT TTA GAG GAA GTA A-3′; ITS2: 5′-GCT GCG TTC TTC ATC GAT GC-3′) were used to amplify the ITS gene of gut fungi. Moreover, PCR was performed as described by Allali et al. (2015) and Sokol et al. (2017). The amplified products were then evaluated by 2% agarose gel electrophoresis, purified with Agencourt AMPure XP beads (Beckman Coulter Co., USA), and quantified using the Qubit dsDNA assay kit. The PCR products were sequenced using an Illumina HiSeq platform (PE250) at Shanghai Oebiotech Co., Ltd., China. The raw data were submitted to the Sequence Read Archive of the NCBI database (https://www.ncbi.nlm.nih.gov/sra) under the accession numbers SRR19633851–SRR19633918 and SRR24782233–SRR24782286. The primers for *Fn, Bb, C. albicans*, and *S. cerevisiae* are listed in Supplementary Table 2. Furthermore, 10 μl SYBR Green II was used as the qPCR system by TAKARA in cooperation with SYBR^®^Premix Ex TaqTMII (TliRNaseH Plus). Stepone^®^plus by ABI company was used in qPCR with all the operation and configuration according to the manufacturer's instruction with 40 cycles of 95°C denaturation for 5 s and 60°C annealing and extension for the 30 s in total after 30 s of pre-denaturation at 95°C.

### Bioinformatics analysis and potential biomarker identification

Raw sequencing data were provided in the Fastq format. Bioinformatics analysis of bacterial 16S rRNA and fungal ITS gene amplicon pyrosequencing data was performed using the Quantitative Insights Into Microbial Ecology (QIIME v.1.8.0) software pipeline, and the combined raw sequencing data were demultiplexed and filtered. Poor quality (below an average quality score of 30) and short sequences (shorter than 200 bp) of all reads were removed using Trimmomatic software (version 0.35). Clean reads were subjected to primer sequence removal and clustered to generate operational taxonomic units (OTUs) using Vsearch software with a 97% similarity cutoff using USEARCH. Alpha diversity indices (Shannon and Simpson) were calculated using Mothur software. Differences between the bacterial and fungal compositions of the two populations were analyzed based on orthogonal partial least squares-discriminant analysis (OPLS-DA) using the mixOmics package in R (v3.2.1). To identify significant differences among the four groups of gut bacteria/fungi, linear discriminant analysis effect size (LEfSe), which performs a non-parametric factorial Kruskal–Wallis rank-sum test followed by the linear discriminant analysis (LDA) coupled with measurements to assess the effect size of each differentially abundant taxon, was carried out through the LEfSe tool with an LDA of 2.0. Functional predictions were made based on 16S rRNA OTU membership using a phylogenetic investigation of communities by reconstruction of unobserved states (PICRUSt), according to the online protocol (http://picrust.github.io/picrust/). Network analyses were performed to categorize the core fungal taxa using OeBiotech tools available at https://cloud.oebiotech.cn/task/ and the Tutool platform at https://www.cloudtutu.com.

### Statistical analysis

Differences in the gut microbial communities between the two groups were analyzed using the non-parametric Kruskal–Wallis rank-sum test. All PCR samples were analyzed in triplicate, and Ct values >5 (Ct_max_ – Ct_min_) or underdetermined readouts were excluded. The average Ct value from triplicates was calculated, and the relative abundance of the target gut microbiota was based on the Ct value, which was defined as the target Ct value minus the Ct value for 16s rRNA. All values are expressed as mean ± standard deviation. A two-tailed *p-*value <0.05 was considered statistically significant. Pairwise multiple comparisons were conducted using analysis of variance (ANOVA), followed by the Bonferroni *post hoc* test. Associations were determined using Spearman's rank correlation. All statistical analyses and associated plots, such as OPLS-DA, pairwise Spearman's correlations, R scores, and *p*-values, were performed using GraphPad Prism v 7.0 and SPSS 22.0.

## Results

### Bacterial composition, diversity analysis, and taxonomic alterations

The 16S rRNA gene was amplified and sequenced from 68 fecal samples from four groups, which were designated as HC (*n* = 15, designated sample numbers HC1–15), while the patient groups included CRA (*n* = 15, sample numbers XH1–15), CRC (*n* = 19, sample numbers WC1–19), and PP (*n* = 19, sample numbers ZL1–19). The sequencing results produced 4,933,539 raw reads. After removing low-quality reads, 4,207,009 clean reads corresponding to 8,501 OTUs were retained. Each sample contained an average of 1,283 OTUs (range, 679–2,407 per sample). In total, 2,629 unique OTUs were identified in the four groups: 482 in the HC, 683 in the CRC, 301 in the CRA, and 1,154 in the PP group. Venn analysis revealed 39 unique core OTUs in 68 samples. Gut bacteria from fecal samples collected from HC comprised 27 phyla, 57 classes, 156 orders, 270 families, and 581 genera; those from CRA included 33 phyla, 73 classes, 182 orders, 298 families, and 623 genera; those from CRC included 34 phyla, 82 classes, 198 orders, 321 families, and 660 genera; and those from PP comprised 33 phyla, 88 classes, 213 orders, 351 families, and 727 genera. The dominant bacterial phyla were Bacteroidetes, Firmicutes, and Proteobacteria, accounting for 48.17, 37.35, and 10.40% of the OTUs, respectively (Supplementary Figure 2A). The dominant bacterial classes were the *Bacteroidia, Clostridia*, G*ammaproteobacteria, Negativicutes*, and *Actinobacteria* (Supplementary Figure 2B). The predominant bacterial orders were *Bacteroidales, Oscillospirales, Lachnospirales, Enterobacterales*, and *Lactobacillales* (Supplementary Figure 2C). The dominant bacterial families were the *Bacteroidaceae, Lachnospiraceae*, Prevotellaceae, *Ruminococcaceae*, and *Enterobacteriaceae* (Supplementary Figure 2D). The dominant bacterial genera in the four groups were *Bacteroides, Prevotella, Faecalibacterium, Muribaculaceae*, and *Lachnoclostridium* (Supplementary Figure 2E).

**Figure 1:**
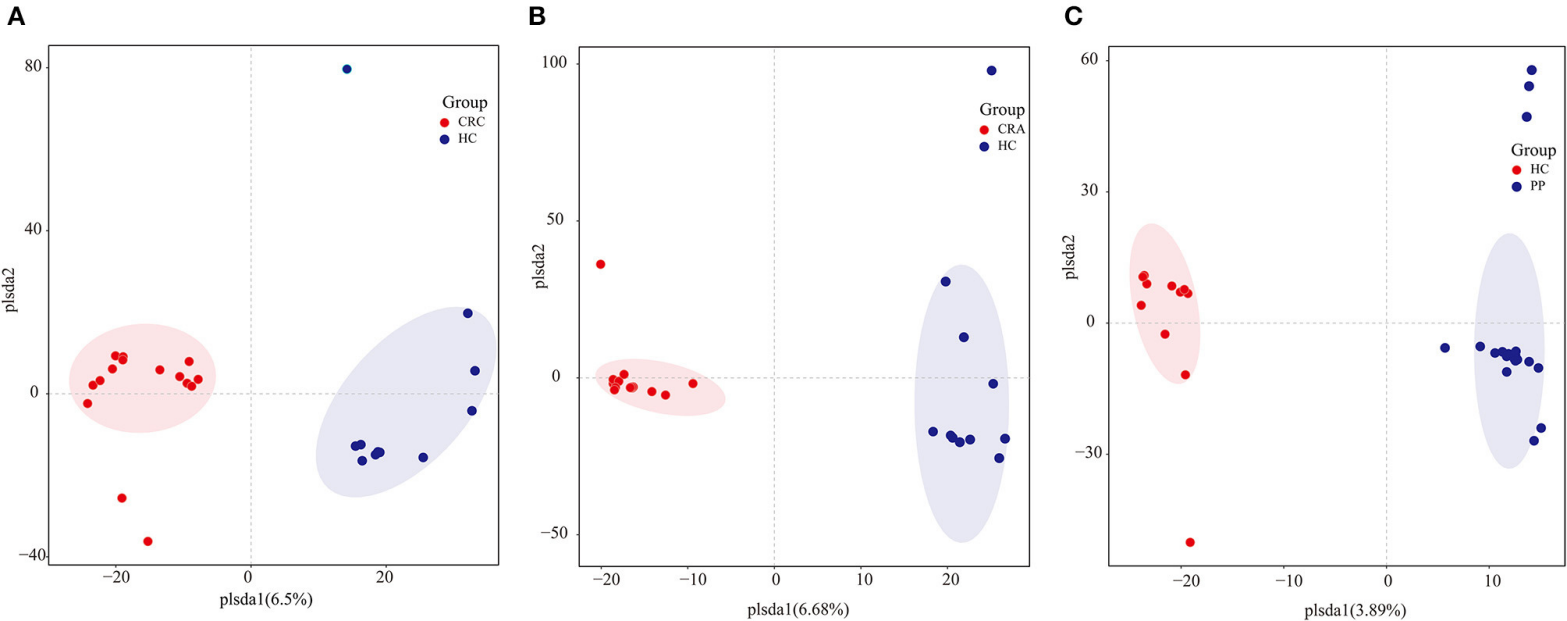
Group-wise comparisons by OPLS-DA analysis of the gut bacteria in HC vs. CRC **(A)**, HC vs. CRA **(B)**, and HC vs. PP **(C)**.

To determine potential shifts in the gut bacterial composition among patients and HC, we compared the alpha and beta diversities among the four groups. However, no changes in alpha diversity (Simpson and Shannon indices) were detected between HC vs. CRC, HC vs. CRA, or HC vs. PP. Moreover, beta diversity comparisons among the samples evaluated by OPLS-DA demonstrated that all CRC samples clustered together, except for CRC2, whereas all HC samples clustered together, except HC8 (Figure 1A). All CRA individuals clustered together, except for CRA1 and CRA2 (Figure 1B), whereas all PP individuals clustered together, except for PP16, PP17, and PP18 (Figure 1C).

**Figure 2:**
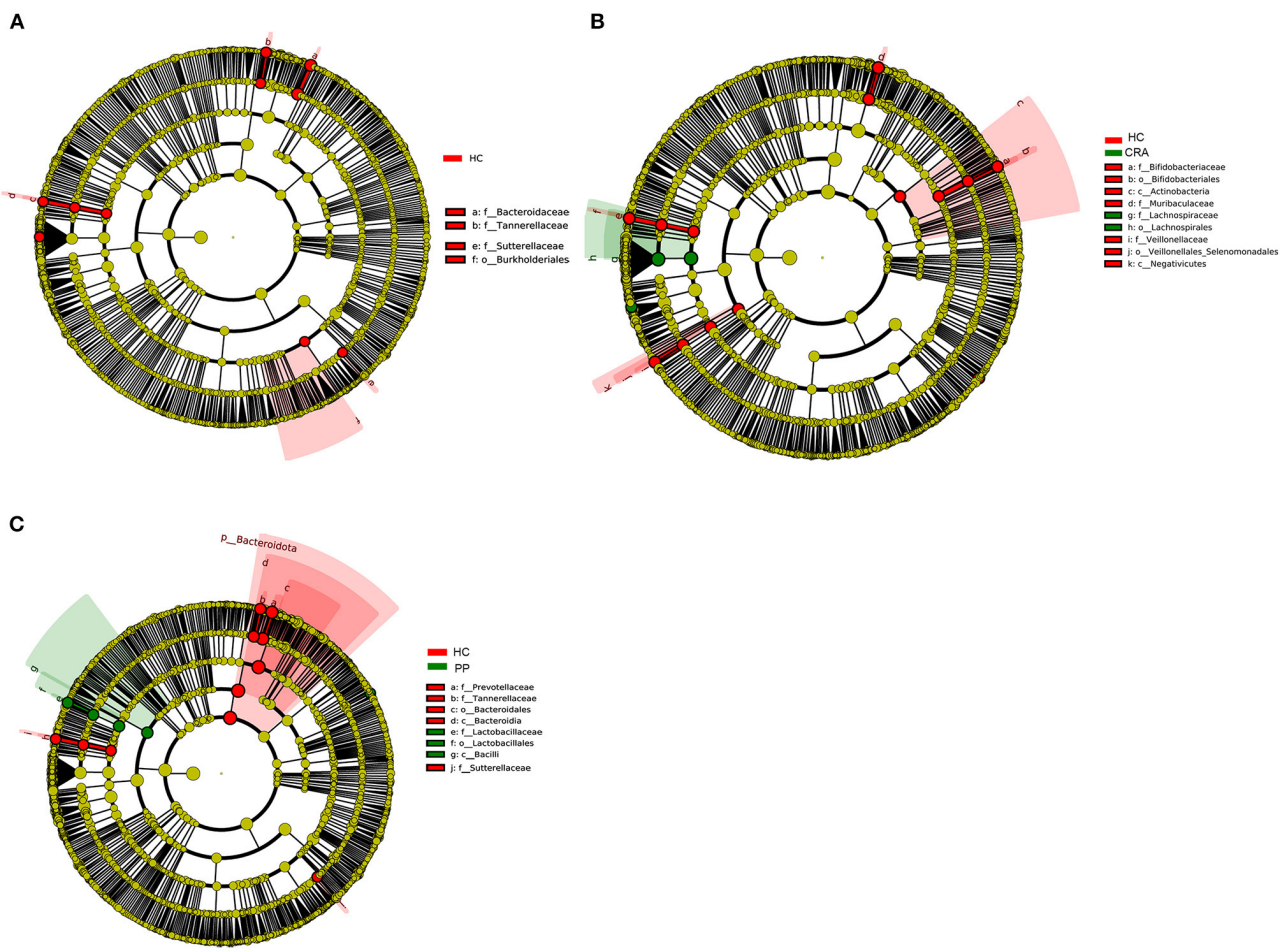
The most differentially abundant gut bacterial taxa in HC vs. CRA **(A)**, HC vs. CRC **(B)**, and HC vs. PP **(C)** based on LEfSe analysis, respectively.

High-throughput sequencing data were analyzed to determine which gut bacteria were significantly associated with the HC or patient groups. A total of 51 species of bacteria were identified in the HC, CRA, CRC, and PP groups. *Bacteroides vulgatus, Bacteroides plebeius, Parabacteroides merdae, Romboutsia ilealis*, and *Sutterella wadsworthensis* were the top five most dramatically different species among the four groups. Additionally, LEfSe was used to determine the taxa that most likely revealed differences between CRA/CRC patients and healthy controls, and *Alphaproteobacteria* were increased in the CRA group (Figure 2A). Comparing the HC with CRC groups, *Subdoligranulum* was increased in CRC while *Bacteroides, Bacteroidaceae, Tannerellaceae* and *Parabacteroides* were increased in HC samples (Figure 2B). Other comparisons revealed that *Firmicutes, Bacill, Lactobacillales, Lactobacillus*, and *Lactobacillaceae* were significantly enriched in the PP group, whereas *Bacteroidales, Bacteroidia, Bacteroidota, Bacteroides*, and *Bacteroidaceae* were more abundant in the HC group than in the PP group (Figure 2C).

In different sample groups, PICRUSt was implemented as a predictive tool for the gut bacterial communities. Overall, 24 Kyoto Encyclopedia of Genes and Genomes (KEGG) orthologs were identified in the KEGG database. Among all samples, PICRUSt analysis indicated that carbohydrate transport and metabolism, transcription, amino acid transport and metabolism, and cell wall/membrane/envelope biogenesis accounted for 11.33, 9.04, 8.42, 7.75, and 6.96% of all functional predictions, respectively (Supplementary Figure 3). Moreover, most of the major functions of the gut bacterial communities in the HC, CRA, CRC, and PP groups (except RNA processing and modification, chromatin structure and dynamics, cell wall/membrane/envelope biogenesis, inorganic ion transport and metabolism, intracellular trafficking, secretion, vesicular transport, defense mechanisms, extracellular structures, and nuclear structure) were significantly different among the four groups.

### Fungal composition, diversity analysis, and taxonomic alterations

The ITS gene region was sequenced and analyzed from the same fecal samples, except 15 samples. A total of 54 fecal samples from four designated groups were collected: healthy subjects were designated as HC (*n* = 14, designated sample numbers HC1–14), while the patient groups included CRA (*n* = 14, sample numbers CRA1–14), CRC (*n* = 11, sample numbers CRC1–11), and PP (*n* = 15, sample numbers PP1–15). A total of 4,017,759 reads were retained, and after removing low-quality reads, 3,271,674 clean reads corresponding to 2,970 OTUs were retained. Each sample contained 60,586 ± 9,877 reads (range, 34,481–69,982 per sample), and an average of 55 OTUs (range, 14–311) and 96.5% (mean) were classified as fungal phyla.

Gut fungi from the fecal samples collected from HC comprised 11 phyla, 27 classes, 58 orders, 123 families, and 186 genera; those from CRA included 8 phyla, 20 classes, 41 orders, 71 families, and 89 genera; those from CRC included 8 phyla, 18 classes, 31 orders, 52 families, and 58 genera; and samples collected from PP comprised 8 phyla, 22 classes, 37 orders, 64 families, and 80 genera. The dominant bacterial phyla were *Ascomycota, Basidiomycota*, and *Glomeromycota*, accounting for 70.59, 25.29, and 1.22%, respectively (Supplementary Figure 4A). The dominant fungal classes were *Saccharomycetes, Agaricomycetes, Eurotiomycetes, Dothideomycetes*, and *Sordariomycetes* (Supplementary Figure 4B). The main fungal orders identified were *Saccharomycetales, Eurotiales, Pleosporales, Hypocreales*, and *Agaricales* (Supplementary Figure 4C). The main fungal families identified were *Thermoascaceae, Nectriaceae, Phaeosphaeriaceae, Mycosphaerellaceae*, and *Saccharomycetaceae* (Supplementary Figure 4D). The main fungal genera in all four groups were *Candida, Byssochlamys, Phaeosphaeria, Gibberella*, and *Mycosphaerella* (Supplementary Figure 4E).

**Figure 3:**
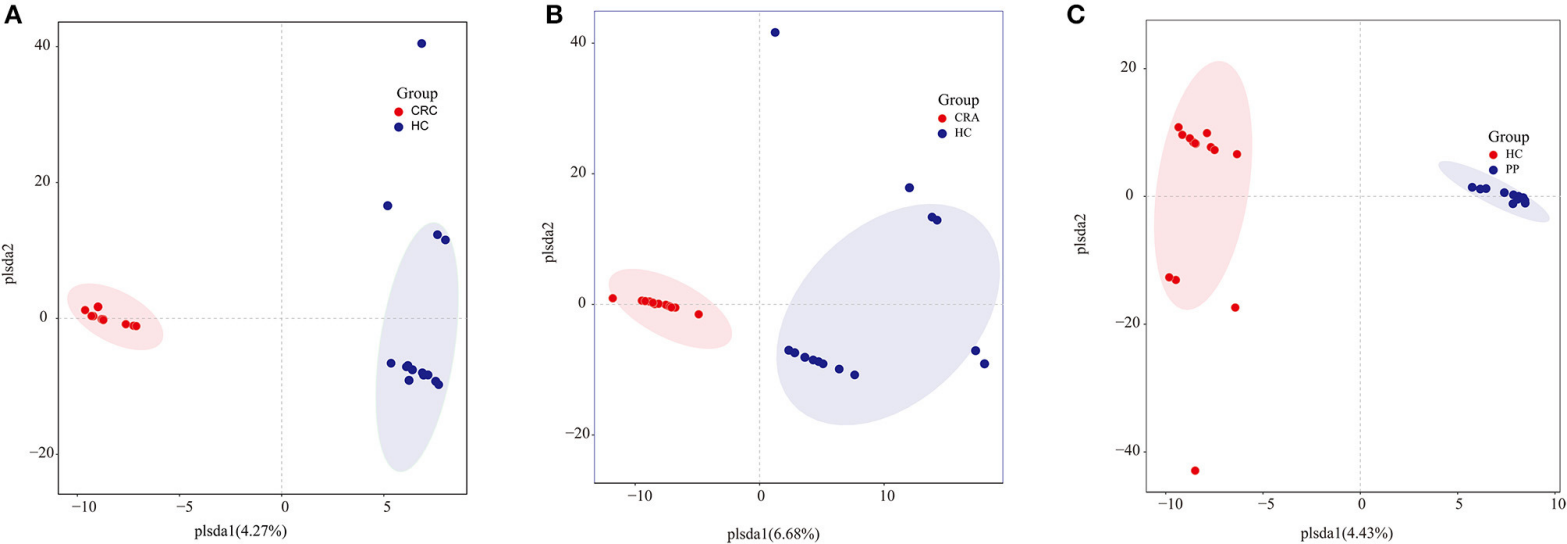
Group-wise comparisons by OPLS-DA analysis of the gut fungi in HC vs. CRC **(A)**, HC vs. CRA **(B)**, and HC vs. PP **(C)**.

Similar to gut bacterial alpha diversity, gut fungal alpha diversity was estimated using the observed Simpson and Shannon indices. The alpha diversity of the HC samples was significantly higher than those of the CRC, CRA, and PP samples, as indicated by the number of observed OTUs. The results exhibited no changes in alpha diversity (Simpson and Shannon indices) among the groups, except for the Shannon indices, which demonstrated that alpha diversity differed significantly between HC and CRC and between HC and PP samples. Moreover, individuals from each group were irregularly distributed according to their clade as identified by OPLS-DA (Figures 3A–C).

**Figure 4:**
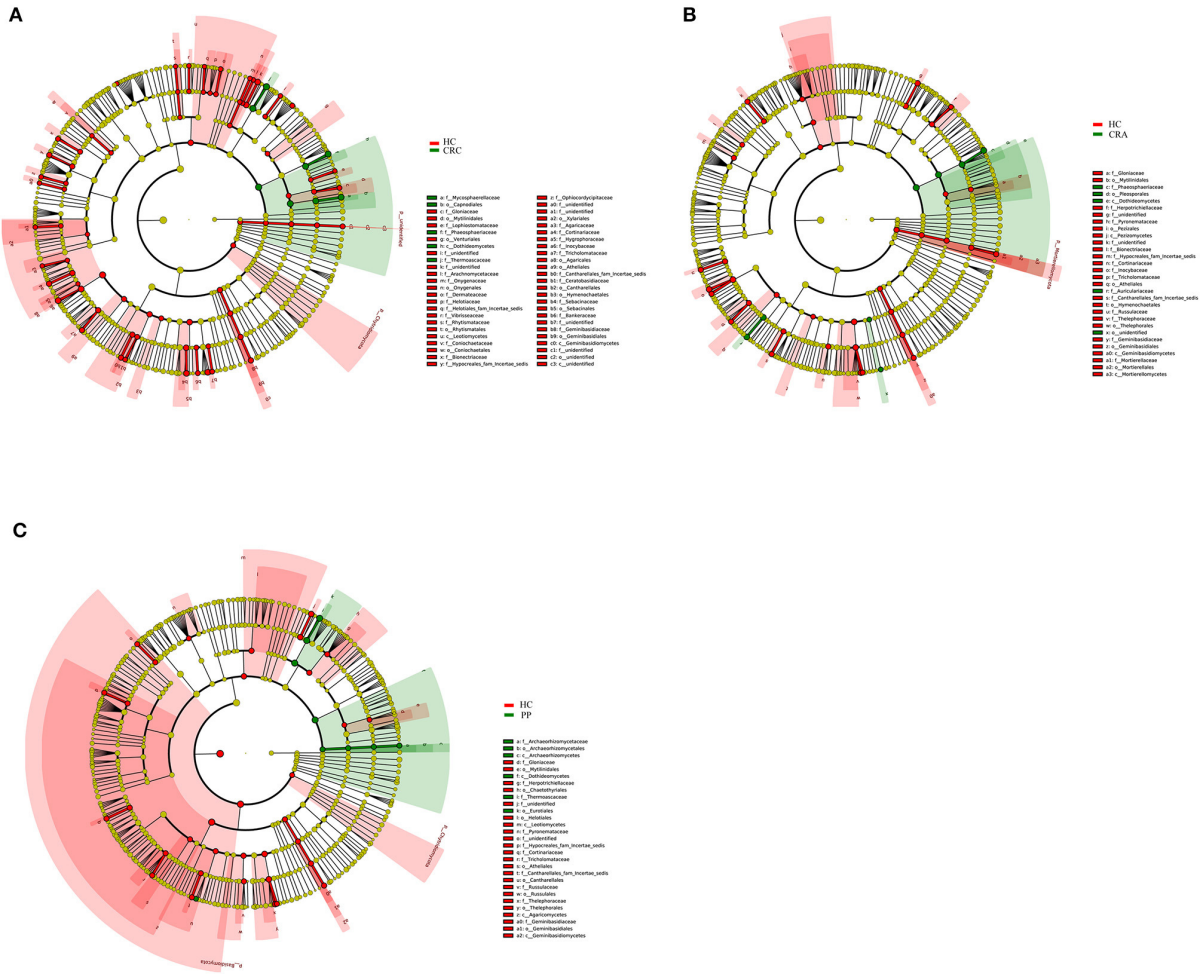
The most differentially abundant gut fungal taxa in HC vs. CRA **(A)**, HC vs. CRC **(B)**, and HC vs. PP **(C)** based on LEfSe analysis, respectively.

To explore the variations in fungal community compositions between the two groups, the relative abundances of several taxa were compared among the four groups using LEfSe analysis (Figures 4A–C). High-throughput sequencing data were analyzed to determine which gut fungi were significantly associated with the HC or patient groups. Eight fungal species were identified in the HC, CRA, CRC, and PP groups. *Byssochlamys spectabilis, Russula sanguinea, Cortinarius bovarius, Geminibasidium hirsutum*, and *Tricholoma bonii* were the top five dramatically different fungal species among the four groups.

### Network analysis, heatmap, and bacteria-fungi associations

We constructed genus-level correlation networks for both gut bacteria and fungi in the HC vs. CRA, HC vs. CRC, and HC vs. PP groups. The results indicated that *Clavaria, Botryotrichum*, and *Olpidium* were the hub fungal genera in each model of the HC vs. CRC (Supplementary Figure 5A). *Cladophialophora* and *Ganoderma* were the hub fungal genera, and *Haemophilus* was the hub bacterial genus in each model of HC vs. CRA (Supplementary Figure 5B). *Ceratobasidium, Cutaneotrichosporon, Parastagonospor*, and *Saitozyma* were the hub fungal genera, and *Mucispirillum* and *Klebsiella* were the hub bacterial genera in each model of the HC vs. PP (Supplementary Figure 5C).

**Figure 5:**
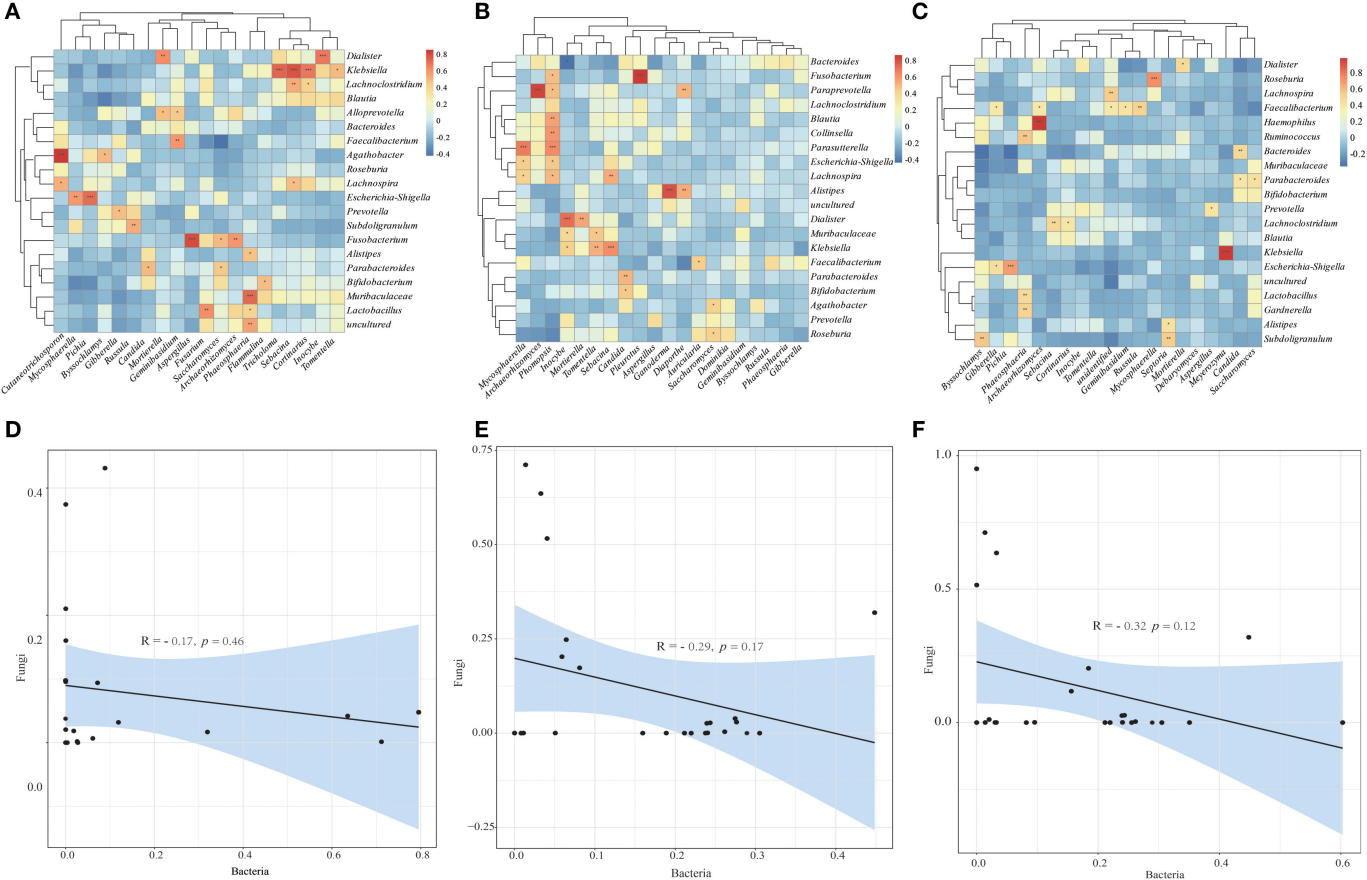
Heatmaps illustrating correlations between the top 20 differential bacterial genera and top 20 differential fungal genera in HC vs. CRC **(A)**, HC vs. CRA **(B)**, and HC vs. PP **(C)**, respectively. Correlation between bacterial and fungal data for HC vs. CRC **(D)**, HC vs. CRA **(E)**, and HC vs. PP **(F)**. *p < 0.05, **p < 0.01, and ***p < 0.001. Red: positive correlation; blue: negative correlation.

To investigate the relationship between bacterial and fungal taxa, we analyzed the correlations between the top 20 bacterial genera and the top 20 fungal profiles. Comparison of HC with CRC samples exhibited positive correlations between the abundance of the *Klebsiella* genera with *Cortinarius, Sebacina* with *Tricholoma, Dialister* with *Inocybe, Cutaneotrichosporon* with *Agathobacter, Fn* with *Aspergillus*; however, there was no negative correlation (Figure 5A). Comparison of HC with CRA samples identified positive correlations between the abundance of *Mycosphaerella* and *Parasutterella, Paraprevotella* and *Archaeorhizomyces, Phomopsis* and *Lachnospira, Escherichia-Shigella, Parasutterella, Collinsella*, and *Blautia* (Figure 5B). *Faecalibacterium* was found to be positively correlated with *Gibberella, Archaeorhizomyces, Russula, Geminibasidium*, and *Klebsiella* with *Meyerozyma* (Figure 5C). We calculated the correlations between bacterial and fungal data for HC vs. CRC, HC vs. CRA, and HC vs. PP comparisons. We established a highly significant negative correlation between the bacterial and fungal data for HC vs. CRC (R = −0.17, *p* = 0.46) (Figure 5D), with similar results obtained between the bacterial and fungal data for HC vs. CRA (R = −0.29, *p* = 0.17; Figure 5E) and HC vs. PP (R = −0.32, *p* = 0.12; Figure 5F).

### qPCR analysis of some microbial species in HC, CRC, CRA, and PP

**Figure 6:**
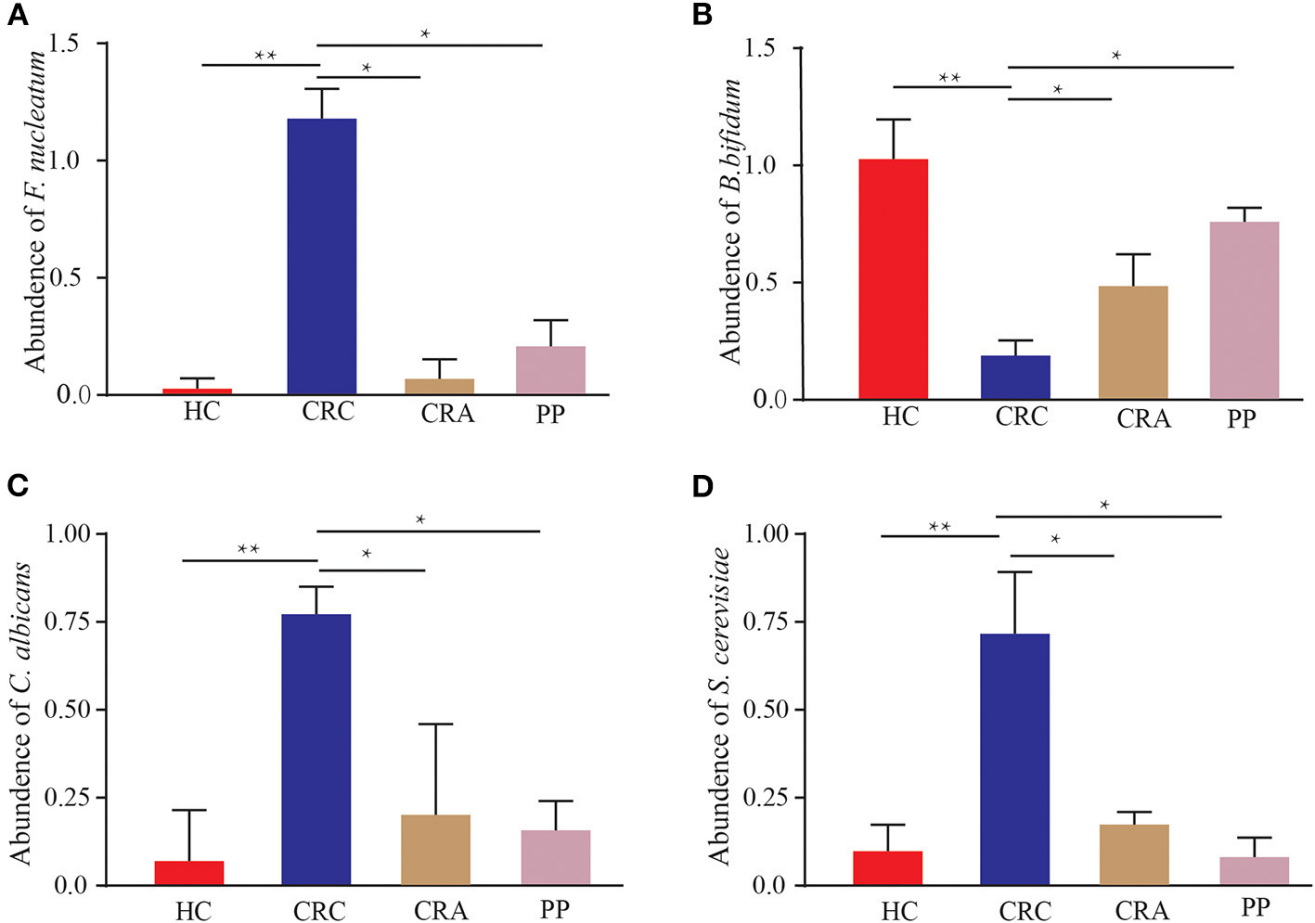
The abundance of *Fusobacterium nucleatum* **(A)**, *Bifidobacterium bifidum* **(B)**, *Candida albicans* **(C)**, and *Saccharomyces cerevisiae* **(D)** in HC, CRC, CRA, and PP, respectively. *p < 0.05 and **p < 0.01.

The 402 patients in the test cohort consisted of 92 HC, 119 CRC patients, 95 CRA patients, and 96 PP. We discovered that *Fn* demonstrated a significantly increasing trend in the feces of CRC patients compared to that in HC (*p* < 0.01); however, the abundant levels in CRA and PP were significantly lower than those in CRC (*p* < 0.05) (Figure 6A). The relative abundance of *Fn* exhibited a mild increase in CRA and PP, which was higher than that in HC, but lower than that in CRC (Figure 6A). Our results indicated the abundance of *C. albicans* and *S. cerevisiae* in CRC, as described in *Fn* (Figures 6C, D). In our cohort, the relative abundance of *Bb* exhibited different results; it demonstrated a statistically significant difference in CRC compared to HC, CRA, and PP. Compared to healthy controls, the relative abundance of *Bb* in HC was higher than that in CRA, CRC, and PP; however, the relative abundance of *Bb* in CRA and PP was higher than that in HC, but lower than that in CRC (Figure 6B).

## Discussion

The incidence of CRC is rising worldwide; sporadic CRC accounts for 95% of all cases, with various pathways, including the adenoma-adenocarcinoma pathway, inflammatory pathway, and *de novo* pathway. Additionally, CRA is regarded as one of the major precancerous lesions of CRC, of which 60–90% arises via the traditional adenoma-carcinoma pathway (Conteduca et al., 2013; Feng et al., 2019; Kim et al., 2020). Genetic and environmental factors play important roles in the pathogenesis of CRC as gut bacteria and fungi contribute to the gut ecosystem through their key roles in host interactions (Bhopal, 2015; Printz, 2015; Yang et al., 2019). Accumulating evidence has revealed that gut dysbiosis is one of the most essential environmental factors in the initiation and progression of CRC, and several studies have indicated that gut bacteria and fungi can act as drivers to initiate precancerous lesions by promoting the accumulation of gene mutations, thereby directly implicating them in the development of CRA and subsequent progression to CRC (Gao et al., 2017; Coker et al., 2019; Liang et al., 2020; Liu et al., 2020). Although colonoscopy is an effective screening tool for CRC diagnosis, it remains unpopular with the subjects being tested. Moreover, studies have increasingly addressed the role of the gut microbiome in CRC since a dysbiotic state has been reported in the gut microbiome of CRA and CRC patients (Allali et al., 2015). Hence, there is an unmet need to develop effective non-invasive examinations to detect the early development of CRC (Feng et al., 2023). High-throughput gene sequencing and metagenomic studies have exhibited decreased overall diversity and increased abundance of certain tumor-promoting bacteria and fungi in tumor tissues (Wang et al., 2012). Our study aimed to test whether the gut microbiome composition of patients with CRC and CRA differs from that of HC. We observed changes in the composition and structure of gut bacteria and fungi in patients with CRC or CRA, similar to previous reports revealing abnormalities in normal bacterial and fungal community composition in the guts of patients with CRC or CRA (Lu et al., 2016; Coker et al., 2019).

The gut microbiome plays complex and key roles in numerous diseases, and a structural imbalance does exist in the gut microbiome of patients. Furthermore, accumulating evidence indicates that the etiology of CRC is related to the gut microbiota (Sheng et al., 2020). Over the last few decades, an increasing number of studies have indicated that the assemblage of the gut microbiota influences the formation and progression of CRC (Geng et al., 2013; Garcia-Castillo et al., 2016; Guthrie et al., 2017). In this study, we investigated the structure of gut bacterial and fungal communities in HC, CRA, CRC, and PP samples. The analysis enabled the elucidation of differences in the guts of CRC, CRA, and PP groups from the HC group. For the bacterial data, alpha diversity analysis did not reveal any difference in the observed species; however, the diversity indicated by the Simpson index in the HC group was higher than that in the CRC group. There was also no significant difference between the CRA and PP groups, which has also been observed in previous studies (Shen et al., 2010; Sanapareddy et al., 2012; Feng et al., 2015; Lu et al., 2016). For fungal data, the Shannon diversity index results established that alpha diversity differed significantly between HC and CRC and between HC and PP. However, beta diversity analyses of HC vs. CRA, HC vs. CRC, and HC vs. PP demonstrated dramatic differences in both bacterial and fungal structures, revealing that the process from CRA to CRC resection had a strong effect on diversity (Shen et al., 2010; Sanapareddy et al., 2012; Feng et al., 2015; Lu et al., 2016). Therefore, this confirmed the perspective that the changes in bacterial and fungal profiles were not entirely elicited by CRA or CRC itself and that bacteria and fungi might play vital roles in CRA or CRC development, while the existing abnormal environment after adenoma resection might also contribute to adenoma recurrence (Yu, 2018). Many studies have confirmed increased microbial diversity in CRC patients compared to HC, suggesting that microbial diversity can be distinguished from HC even when the CRC is excised (Yu, 2018). It was discovered that not only the removal of CRC provided gut microbiota with great alterations but also these alterations encouraged a more normal microbiota, confirming the view that the gut microbial community was not entirely produced by CRC itself and might play a vital role in CRC formation, while the still existing abnormal environment after CRC resection might also lay the groundwork for CRC recurrence (Sze et al., 2017).

Bacteria and fungi are important members of the gut microbial ecosystem that interact with the host, and many factors, such as age, sex, and types of cancer, affect the diversity and composition of the gut mycobiota; however, limited evidence has been proposed that relies on a high-throughput platform to explore the role of bacteria and fungi in CRC development and impact treatment progression (Gao et al., 2017; Rahwa et al., 2020). Some microbial pathogens directly promote CRC progression, and some microbial metabolites may reduce the risk of CRC. Previous research has established that gut bacteria and fungi are greatly altered in patients with the removal of colorectal CRA/CRC, with post-operative gut bacteria characterized by reductions in commensal bacterial species and the growth of detrimental bacterial and fungal strains (Keku et al., 2015; DeGruttola et al., 2016; Yu et al., 2017). In this study, we identified 51 species of bacteria and 8 species of fungi with significantly altered abundance, especially 2 bacterial species (*Fn* and *Bb*) and 2 fungal species (*C. albicans* and *S. cerevisiae*). A considerable number of studies have identified *Fn* as a potential marker for CRC detection, and it is one of the most widely studied bacteria associated with CRC (Yu et al., 2017). As an obligate anaerobic gram-negative bacillus, *Fn* commonly colonizes the oral cavity, and it has also been detected in CRC and CRA (Castellarin et al., 2012). A previous study identified that *Fn* was abundant in tissues of CRC patients with recurrence after chemotherapy and was associated with clinicopathological characteristics. Enriched *Fn* in CRC has an invasive role in colonic epithelial cells. Furthermore, bioinformatic and functional studies have demonstrated that *Fn* promotes colorectal cancer resistance to chemotherapy (Yu et al., 2017). Among the various fecal microbiological tests for CRC diagnosis, qPCR testing for fecal *Fn* abundance exhibits the potential for popularization and may serve as a possible indicator of CRC prognosis (Suehiro et al., 2016). In this study, we discovered that radical surgery may lead to a rapid decline in fecal *Fn* abundance. As for the role of *Fn* in CRC prognosis, it was discovered that CRC patients with higher *Fn* count had a poor prognosis, suggesting its potential value as a non-invasive prognosis indicator for CRC. Some studies have displayed that fecal *Fn* plays a vital part in CRC prognosis and seems to be firmly linked to its treatment response (Flanagan et al., 2014). Despite accumulating achievements in the non-invasive microbial diagnosis of CRC, few studies have investigated cancer prognosis, and most studies are limited to tests relying on *Fn*. Due to the negative correlation between the abundance and survival of *Fn*, it may serve as a promising prognostic indicator for CRC (Mima et al., 2015; Yamaoka et al., 2018). In humans, *Bb* is distributed across seven different ecological niches, including the gastrointestinal tract and oral cavity, and displays notable physiological and genetic features encompassing adhesion to epithelia as well as the metabolism of host-derived glycans (Turroni et al., 2019). Preclinical reports have displayed that *Bb* can be applied as a biotherapeutic agent in the inhibition or therapy of colorectal cancer through the modification of gut bacteria, and it can play a relevant role in inhibiting colon cancer cell growth, which can be used to prevent some incurable diseases such as cancer (Agah et al., 2019). Previous studies have indicated that the oral consumption of *Bb* probiotics significantly decreased the levels of triglycerides, alkaline phosphatase, low-density lipoprotein, VDR, and LPR gene expression in mouse colon cancer (Tjalsma et al., 2012; Feng et al., 2015; Yang et al., 2019; Kim et al., 2020; Vacante et al., 2020). *C. albicans* has emerged as a major public health problem over the past two decades. The spectrum of diseases caused by *Candida* species ranges from vaginal infections to deep infections in hospitalized patients, which leads to high morbidity and mortality rates and may also play a role in the persistence or worsening of some chronic IBDs (Poulain, 2015). The association between *C. albicans* and cancer has been observed for decades, and most of the current clinical and animal evidence supports *C. albicans* as a member of the oral microbiota that acts as an opportunistic pathogen, along with changes in the epithelium that can predispose individuals to pre-malignancy and/or malignancy (Febriyanti et al., 2022). *S. cerevisiae* is a ubiquitous yeast widely used in industry, and it is also a common colonizer of human mucosae; however, the incidence of invasive infection by these fungi has significantly increased in recent decades (Souza et al., 2013). *S. cerevisiae* has been a key experimental organism for the study of infectious diseases, it has revealed that the abundance of *S. cerevisiae* decreased, while that of *C. albicans* increased in CRC, and a shift in the gut microbiota environment was demonstrated by analyzing the correlation between bacteria and fungi (Coker et al., 2019). We identified CRA-, CRC-, and PP-specific shifts in bacterial and fungal composition by qPCR analysis reflected by the enrichment of *Fn, Bb, C. albicans*, and *S. cerevisiae*. The shift has been previously highlighted, suggesting that the aforementioned species may constitute “biomarker” bacteria associated with cancer-predisposing CRA or outright CRC.

In conclusion, omics initiatives have reached the forefront of biomedical research by highlighting the significance of biological functions and processes. Thus, multi-omic profiling has yielded important insights into CRC biology by identifying potential biomarkers and therapeutic targets. In this study, the gut bacteria and fungi were altered in affected patients compared to those in normal subjects. Thus, our results identified several gut bacteria and fungi that could act as potential “biomarkers” in the traditional adenoma-carcinoma axis using bacterial and fungal data. However, we acknowledge some limitations to our study. The sample numbers were relatively small; therefore, the putative biomarkers, bacteria/fungi, require further validation. This necessitates alternative, larger cohorts to further validate our findings. However, as no single biomarker screen can provide definitive evidence, the findings of this study make significant contributions to the field. Moreover, diet is an important factor to be considered in associating specific fungi with diseases and may affect their universal application as diagnostic markers. The gut microbiota are significantly influenced by food colonization. Further studies are required to identify the functional consequences of the altered bacterial/fungal composition on metabolism and CRC tumorigenesis in the host.

## Data availability statement

The raw data were submitted to the Sequence Read Archive at the NCBI database (https://www.ncbi.nlm.nih.gov/sra) under accession numbers SRR19633851-SRR19633918 and SRR24782233-SRR24782286.

## Author contributions

XL, GL, and FW designed the experiments of this manuscript. JF, ZW, and XL conducted the sample collection and data analysis. JF, XL, and GL wrote the manuscript. All authors read and approved the manuscript.
