# Supplementary material for: Features of combined gut bacteria and fungi from a Chinese cohort of colorectal cancer, colorectal adenoma, and post-operative patients
Source: Front Microbiol. 2023 Aug 8;14:1236583. doi: 10.3389/fmicb.2023.1236583 (PMC10443710; doi:10.3389/fmicb.2023.1236583)
Supplement: Supplementary file 1 [file Data_Sheet_1.pdf]

Supplementary information

Supplementary Tables

Supplementary Table 1. Demographics of the study participants (Mean ± Standard Deviation)

Cohort	Factors	CRC	CRA	PP	HC	*p*-value
Cohort 1	Size	15	19	19	18	
	Age	62.05 ± 12.58	55.42 ± 10.47	55.80 ± 13.4	48.04 ± 5.82	$p = 0.09$
	Gender	Male: 9 Female: 6	Male: 14 Female: 5	Male: 14 Female: 5	Male: 8 Female: 10	$p = 0.52$
	BMI	23.08 ± 3.42	24.11 ± 4.12	23.87 ± 4.22	24.11 ± 3.02	$p = 0.32$
Cohort 2	Size	92	119	95	96	
	Age	62.19 ± 6.21	60.11 ± 4.77	61.11 ± 5.70	62.11 ± 5.32	$p = 0.19$
	Gender	Male: 49 Female: 43	Male: 55 Female: 64	Male: 46 Female: 51	Male: 49 Female: 47	$p = 0.42$
	BMI	23.81 ± 3.34	23.22 ± 3.16	24.51 ± 3.48	24.11 ± 3.38	$p = 0.35$

Supplementary Table 2. qPCR primers and their sequences in this study

Species	Upper primer sequence (5'-3')	Lower primer sequence (5'-3')
Fusobacterium nucleatum	CAACCATTACTTTAACTCTACCATGTTCA	GTTGACTTTACAGAAGGAGATTATGTAAAAATC
Bifidobacterium bifidum	CTTACTTCGCCTTCTTTGCTCCGTAC	AGAAGTCCAAGACTTTGGCCCTGA
Candida albicans	TTTATCATCTTGTCACAGCAGA	ATCCCGCCTTGCCACTACCG
Saccharomyces cerevisiae	AGGAGTGCGGCTCTTAG	TACTTACCGAGGCAAGCTCCA

Supplementary Figures

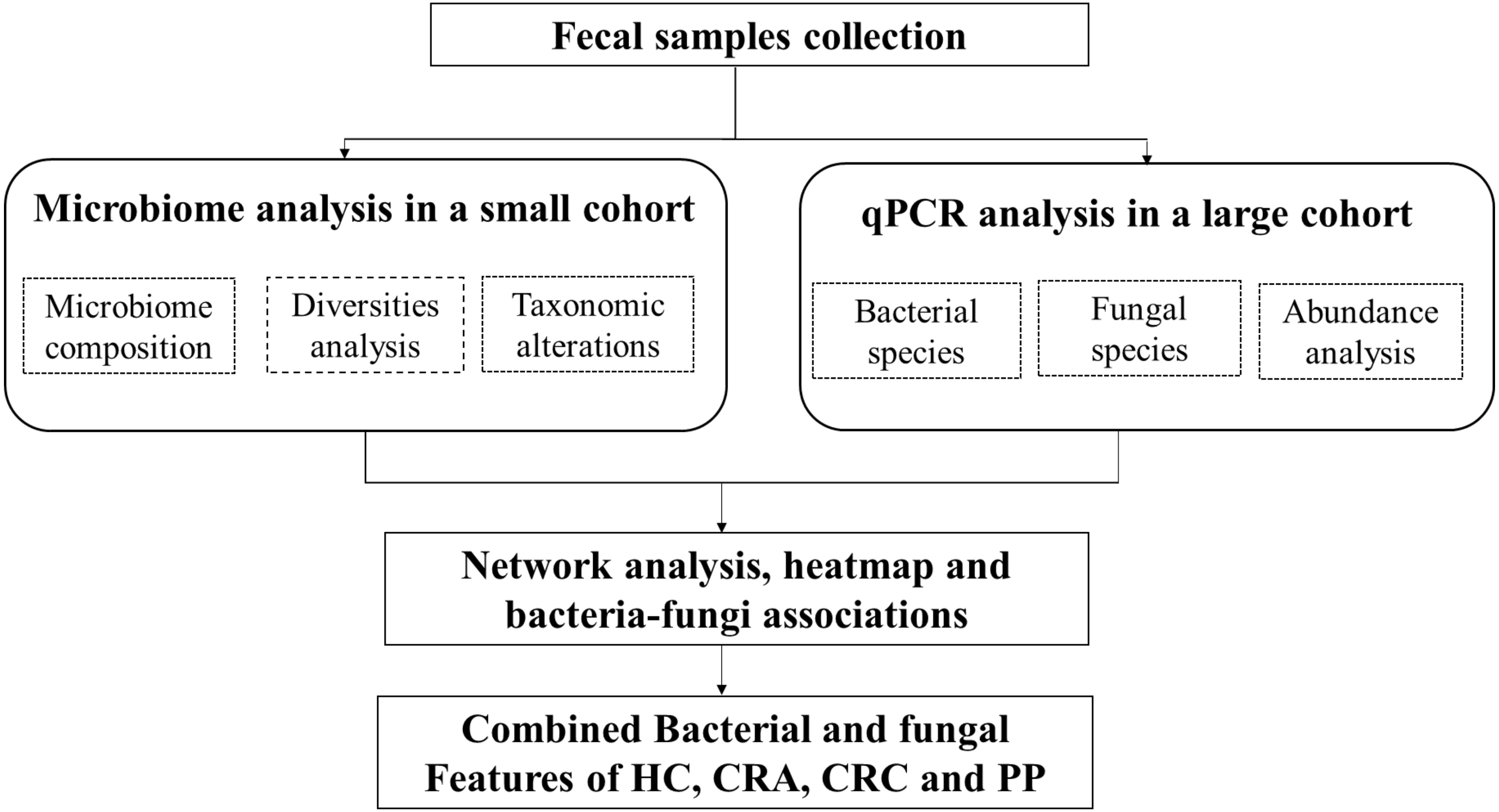

Supplementary Figure 1. Analysis flowchart of the study

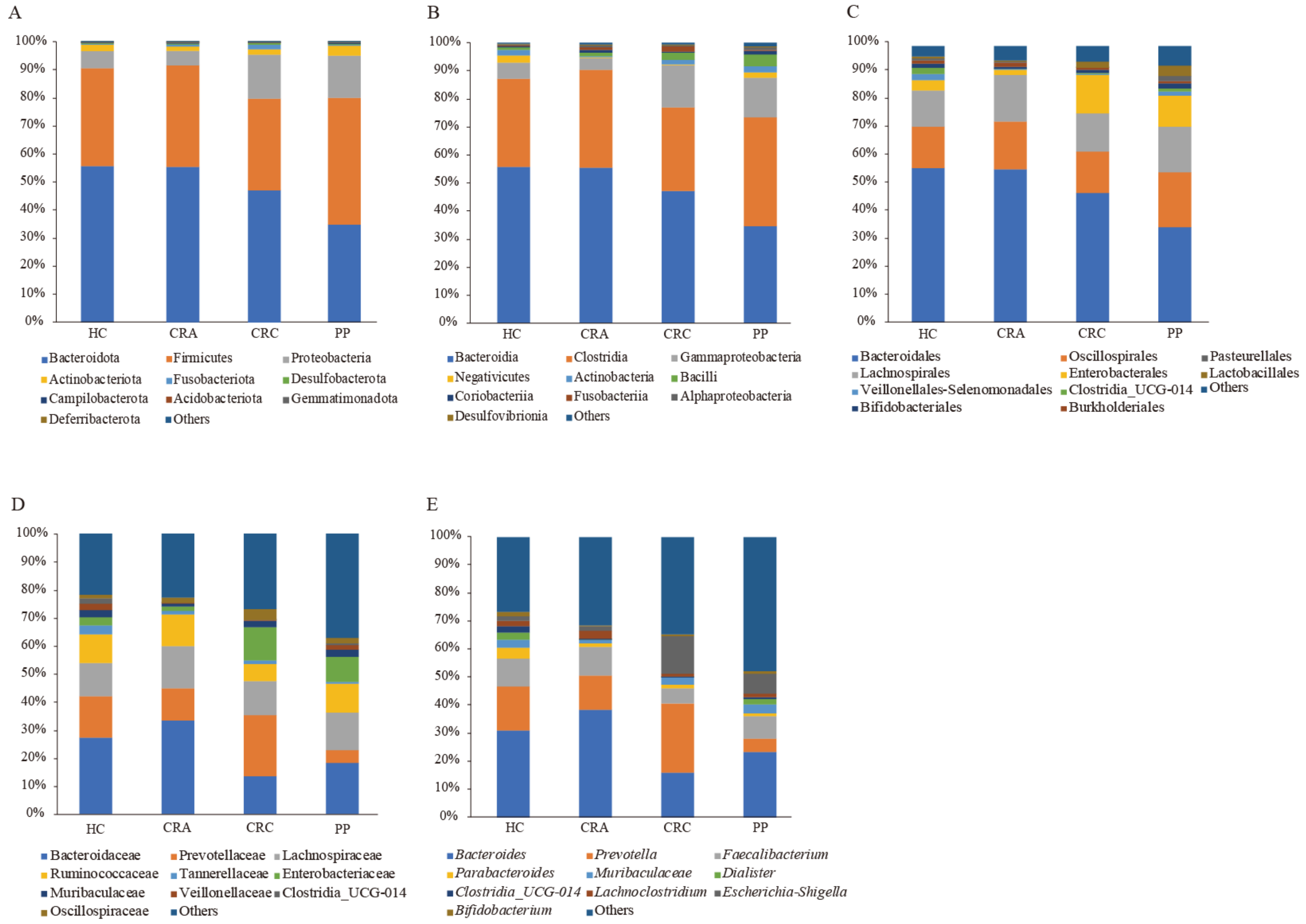

Supplementary Figure 2. Taxonomic analysis of gut bacteria from 16S rRNA sequencing. Compositions of gut bacteria of HC, CRA, CRC, and PP groups at phyla (A), classes (B), orders (C), families (D), and genera (E) levels.

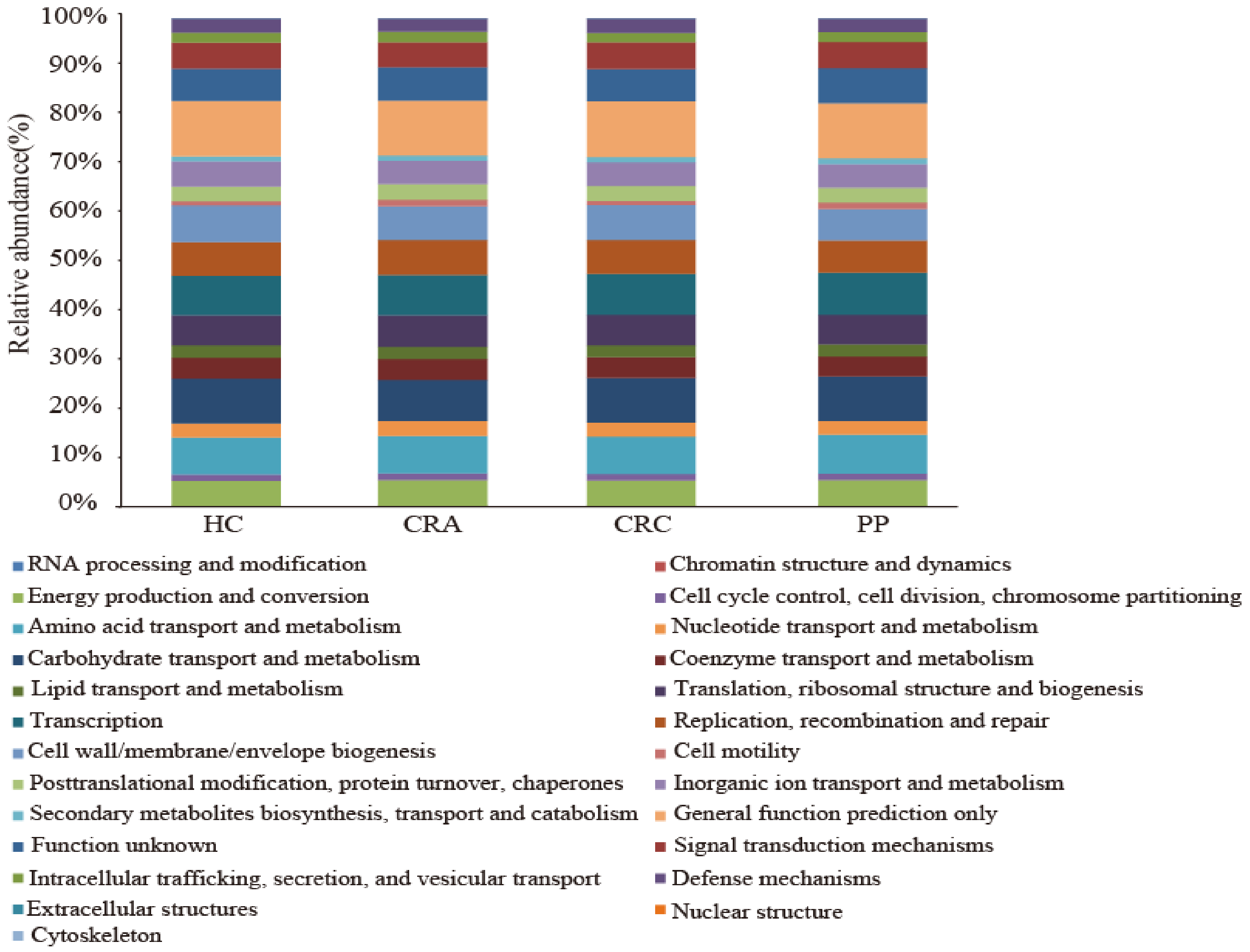

Supplementary Figure 3. Functional predictions for four groups based on PICRUSt analysis.

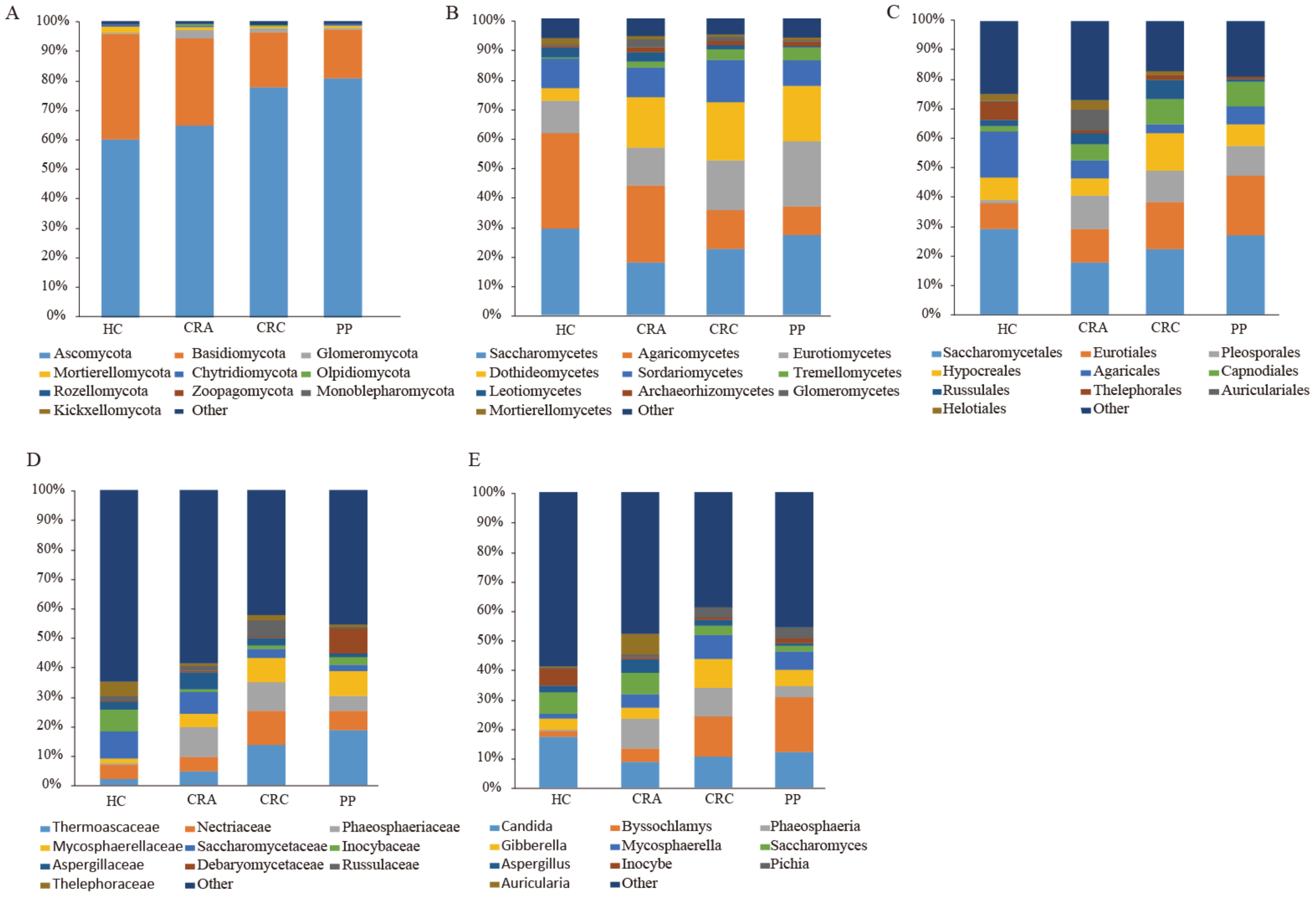

Supplementary Figure 4. Taxonomic analysis of gut fungi from ITS sequencing. Compositions of gut fungi of HC, CRA, CRC, and PP groups at phyla (A), classes (B), orders (C), families (D), and genera (E) levels.

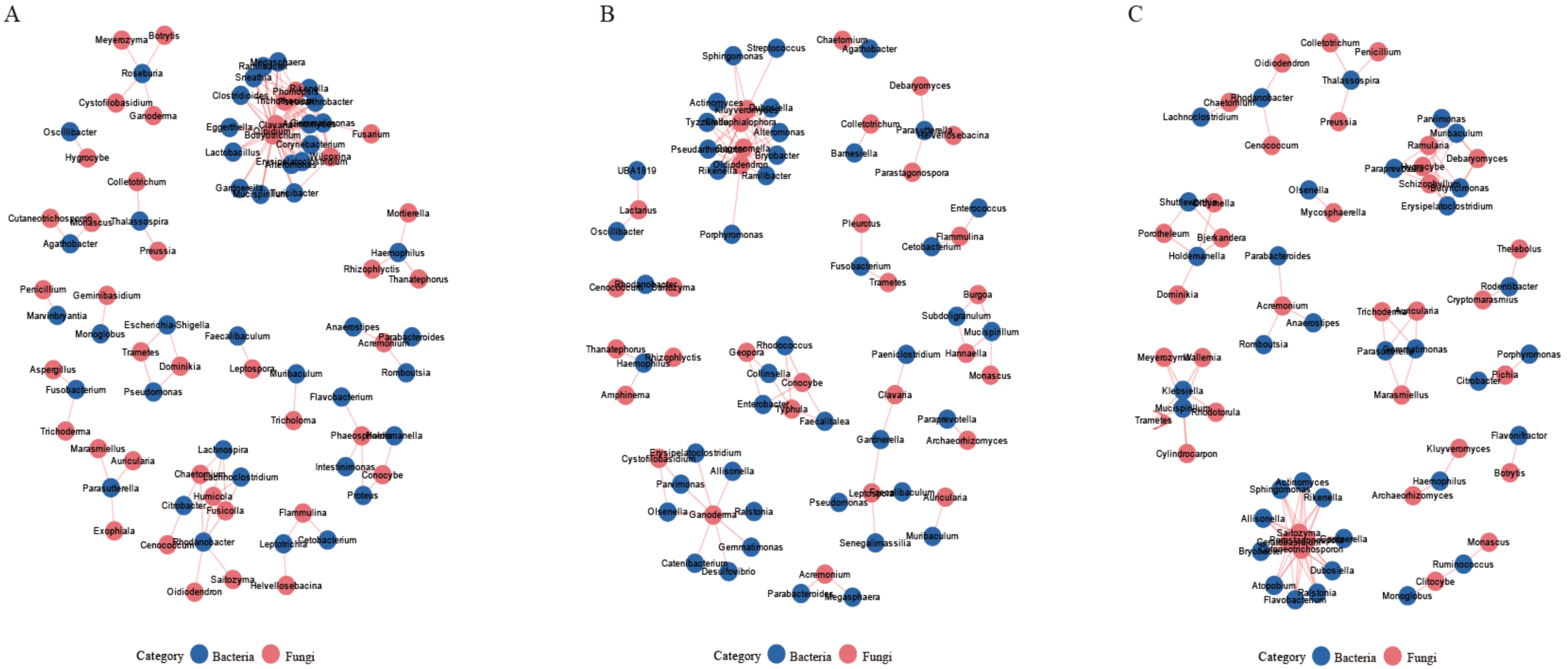

Supplementary Figure 5. Correlation network between bacteria and fungi in HC *vs.* CRC (A), HC *vs.* CRA (B), and HC *vs.* PP (C), respectively. Notes: blue circles: bacteria; red circles: fungi.
